# Tooth loss, diet quality, and cognitive decline: A 15-year longitudinal study

**DOI:** 10.1016/j.jnha.2025.100620

**Published:** 2025-06-27

**Authors:** Lewis Winning, Danielle Logan, Claire T. McEvoy, Dominic Farsi, Gareth J. McKay, Christopher C. Patterson, Peter Passmore, Clive Holmes, Gerard J. Linden, Bernadette McGuinness

**Affiliations:** a Dublin Dental University Hospital, Trinity College Dublin, Lincoln Place, Dublin 2 D02 F859, Ireland; b Centre for Public Health, School of Medicine, Dentistry and Biomedical Sciences, Queen’s University Belfast, Institute of Clinical Sciences, Royal Victoria Hospital, Belfast BT12 6BA, United Kingdom; c Department of Nutritional Sciences, King’s College London, King's College London, Strand, Westminster WC2R 2LS, United Kingdom; d University of Southampton, University Road, Southampton SO17 1BJ, United Kingdom

**Keywords:** Tooth loss, Diet quality, Cognitive decline, Mild cognitive impairment, Dementia

## Abstract

**Objectives:**

To investigate the association between tooth loss and the incidence of mild cognitive impairment (MCI) and dementia, focusing on diet quality as a potential mediator.

**Design:**

Prospective cohort study.

**Setting and participants:**

Community-dwelling older men enrolled in the Northern Ireland PRIME study (Prospective Epidemiological Study of Myocardial Infarction).

**Measurements:**

A group of men, aged 58–71 years, underwent dental examinations in 2001–2003. Surviving participants were invited to attend a cognitive rescreening in 2016–2020, which included a food frequency questionnaire. Cognitive outcomes were categorised as normal, MCI or dementia, based on standardised neurocognitive tests. Dietary diversity scores (DDS) were calculated based on the frequency of intake of food groups. Analyses included multiple logistic regression to evaluate the associations between tooth loss, defined as <20 remaining natural teeth, and MCI/dementia incidence adjusting for potential confounders. Mediation analysis assessed the role of diet quality in this relationship.

**Results:**

A total of 628 men were included, with a mean baseline age of 63.2 years (SD 2.8) and a median follow-up of 15.0 years (IQR 14.2–17.0). At rescreening, 485 (77.2%) were cognitively normal, 112 (17.8%) had MCI, and 31 (4.9%) had dementia. In fully adjusted models, tooth loss was significantly associated with MCI/dementia (OR = 2.06, 95% CI 1.20–3.55, *p* < 0.01). DDS partially mediated this relationship, explaining approximately 23% of the total effect.

**Conclusion:**

In this cohort of older men, tooth loss was associated with incidence of MCI/dementia over 15 years, and diet diversity partially mediated this association. These findings highlight an important pathway connecting oral health, dietary quality, and cognitive outcomes.

## Highlights

• Tooth loss doubled the 15-year MCI/dementia risk in older Northern Irish men.
• Dietary diversity explained 23% of the tooth loss–cognition link.
• Denture use suggested a protective trend but was not statistically significant.

## 1 Introduction

Dementia is a major public health issue among aging populations, driven by a complex interplay of genetic, lifestyle, and physiological risk factors [1]. In 2021, over 55 million people worldwide were living with dementia, with nearly 10 million new cases emerging each year [2]. Projections indicate that this number will rise to approximately 153 million by 2050, primarily due to population growth and aging [3]. Mild cognitive impairment (MCI), represents a transitional state between normal aging and dementia [4]. Prevalence of MCI among community-dwelling older adults, aged >70 years, has been reported as high as 22% [5]. Both mild cognitive impairment and dementia are characterised by cognitive decline from a previously attained cognition level [6]. With no current therapeutic options to reverse MCI/dementia, there is an emphasis on prevention through identification of modifiable risk factors utilising a life course approach [1].

Tooth loss is a prevalent condition in aging populations and has been increasingly recognised as a potential risk factor for cognitive decline and dementia [7,8]. Evidence, based mainly on longitudinal cohort studies, has shown that greater tooth loss is associated with worse cognitive outcomes over time [9]. A recent meta-analysis based on eighteen cohort studies and 356,297 participants reported that tooth loss accelerated cognitive decline and was associated with a 20% increased risk of dementia (95% CI: 1.14–1.26) [10]. Despite the evidence, however, existing studies face methodological shortcomings, including insufficient exposure–outcome assessments that do not thoroughly address key mediators [11].

Amongst purported mediators, change in diet quality has emerged as a potential pathway through which tooth loss may influence cognitive decline and dementia risk [12]. Tooth loss commonly leads to reduced masticatory efficiency and poor diet quality by limiting intake of nutrient-dense foods such as fruits and vegetables [13], and nutrients for example, omega-3 fatty acids, B-vitamins, antioxidants, and polyphenols, that are important in preventing cognitive deterioration [[14], [15], [16], [17]]. However, despite the rationale for a proposed mediation pathway these relationships have primarily been studied in isolation, and the potential mediating role of diet quality in the tooth loss-cognition pathway has yet to be thoroughly investigated.

The present study, therefore, seeks to address this gap by investigating the relationship between tooth loss and MCI/dementia risk among a group of men from Northern Ireland, through a 15-year prospective cohort study with a particular emphasis on the mediating role of diet quality.

## 2 Materials and methods

### 2.1 Study subjects

Subjects investigated in this study were participants in the PRIME study (Prospective Epidemiological Study of Myocardial Infarction), which is a longitudinal cohort study of cardiovascular disease among men in Northern Ireland [18]. From 1991 to 1994 2748 men were recruited from local industry, the civil service and general medical practices. The sample represented ∼5% of 50–60 year-old men in the greater Belfast region and broadly matched the social class structure of the population [19]. Between 2001 and 2003, (considered baseline for the current study), the surviving men were recontacted by post and invited to attend a rescreening visit as part of their continuing involvement in the PRIME study. A dental examination was completed for 1558 of these men. Participants also completed questionnaires gathering information on their medical history, social circumstances, demographic background and tobacco use. A physical examination assessed anthropometric measures. Participants additionally undertook a Mini Mental State Examination (MMSE) to assess global cognitive function.

**Fig. 1:**
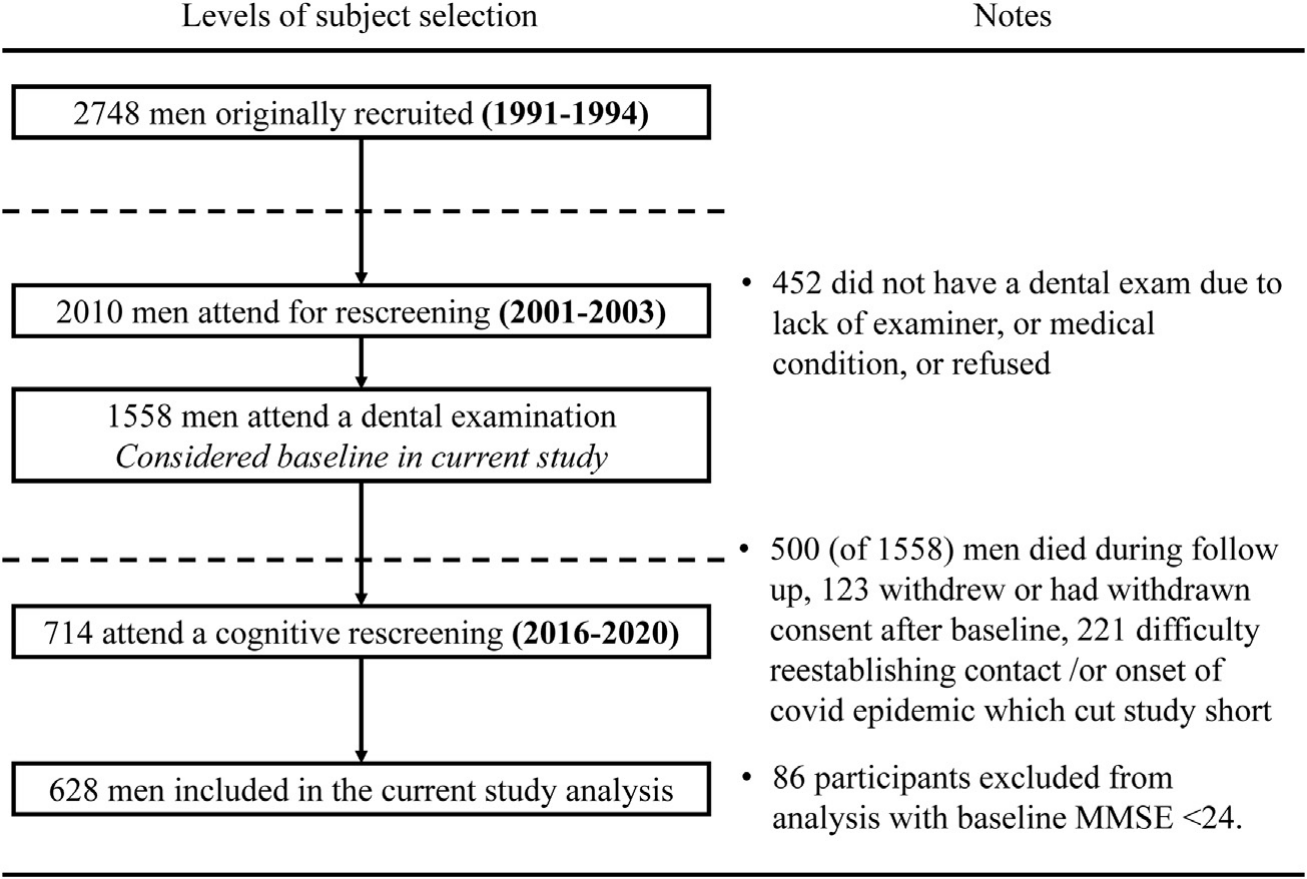
Recruitment and enrolment of study participants flowchart.

Between 2016 and 2020, surviving men were invited to complete a cognitive rescreening (PRIME-COG), where a range of cognitive tests were performed (detailed below). A total of 714 of the 1558 men that had previously had a dental assessment in 2001–2003 attended for the cognitive rescreening in 2016–2020. To reduce the effects from reverse causality participants with a formal diagnosis of any cognitive impairment at baseline examination in 2001–2003 along with men who scored < 24/30 in their baseline MMSE screening were excluded. This resulted in *n* = 628 that were included in the analysis in the current study (Fig. 1). A dietary assessment in the form of a short food frequency questionnaire (FFQ) was completed as part of the PRIME-COG follow up.

Approval for the project was obtained from the Research Ethics Committee of the Faculty of Medicine, Queen's University, Belfast and the Office for Research Ethics Committees (Northern Ireland); reference number 06/NIR02/107, substantial amendment 3. All participants provided informed, written consent. The study was compliant with the Declaration of Helsinki guidelines.

### 2.2 Exposure variable

The main exposure variable for the current study was number of natural teeth at baseline (2001–2003), dichotomised as <20 teeth or ≥20 teeth based on the World Health Organisation definition of a functional dentition [20]. The concept of a functional dentition is particularly relevant in the context of this study, as it directly impacts the ability to consume a diverse and nutritionally adequate diet, a key factor in the mediation pathway being investigated. All dental assessments were completed by one of four dental hygienists who had been calibrated against a “gold standard” set by a senior clinical researcher (G. J. L.) prior to the study. Tooth presence was recorded for each of the 32 teeth to calculate the number of natural teeth present. The examiners used clinical judgement regarding tooth morphology and took into account the respondent’s previous dental history if doubt existed as to the correct notation for a particular missing tooth.

### 2.3 Outcome variable

The main outcome variable for the current study was a consensus diagnosis of normal cognition, MCI, or dementia at the 2016–2020 PRIME-COG follow up. This was based on a repeat MMSE test (previously assessed at baseline 2001–2003) to assess change in global cognition and additional assessment which included domains of attention/executive function, memory, language and visuospatial function using the Addenbrooke’s Cognitive Examination Revised (www.neura.edu.au). Function was assessed using the Bristol Activities of Daily Living Scale (BADLS) [21] and mood/depression assessed using the Geriatric Depression Scale (GDS) short form [22]. The cognitive re-evaluations were performed by research nurses experienced in the use of neuropsychological tests from the Northern Ireland Dementia Clinical Research Network. Following this, and based on the comprehensive cognitive reassessment a consensus diagnosis of: normal cognition for age; MCI; or dementia was made using accepted clinical criteria [[23], [24], [25]], by three experienced clinical researchers (BMcG, APP, CH) who specialize in dementia/AD research.

### 2.4 Mediator variable

The mediation variable used in the current study was diet quality in the form of a diet diversity score (DDS) [26]. As part of the PRIME-COG follow up (2016–2020), a dietary assessment was performed in the form of a short FFQ. The FFQ indicated how often, on average, participants ate specified amounts of food during the past year. There were eight food groups with the portion shown in brackets, including: olive oil/rapeseed oil (1 tablespoon), fruit and natural fruit juice (80 g), vegetables (80 g), oily fish (140 g), wine (125 ml), red meat (1 medium serving), wholegrains (1 medium serving) and nuts (1 small handful). Participants responded with one of the following nine options: never or less than once a month, 1–3/month, once/week, 2–4/week, 5–6/week, once a day, 2–3/day, 4–5/day and 6+/day. A DDS was calculated based on the frequency of consumption of the eight food groups. Participants consuming the food groups at least once a week were assigned a value of one, and a value of zero otherwise. The individual food group scores were summed together to give an overall DDS, ranging from 0 to 8. A higher DDS represented a better dietary diversity and variety of foods in the diet.

### 2.5 Covariates

Covariates with theoretical, practical or previous empirical evidence of association with tooth loss or cognitive decline were included. Age was used as a continuous variable. Baseline cognitive function, as previously mentioned, was assessed using the MMSE and included as a covariate in statistical models. Anthropometric measurements included total waist circumference (to the nearest 0.5 cm); abdominal obesity was defined as a waist circumference ≥94 cm [27]. Participants were classified as current, former or never smokers. Alcohol consumption was categorised according to weekly intake, with those consuming more than 14 units per week classified as exceeding guideline levels. Hypertension was by self-report of the condition in response to the question 'Have you ever been told by a doctor that you have high blood pressure (hypertension)?'. Similarly, diabetes was also by self-report of the condition. Atherosclerotic cardiovascular disease (ACVD) was recorded for men who had a previous diagnosis of coronary heart disease, ischemic cerebrovascular disease or peripheral arterial disease. Accurate information pertaining to ACVD was available from the main study database (as this is the primary outcome under investigation in the PRIME study, with men having already been under observation for 10 years prior to entry to the current study) [28]. Socio-economic status (material conditions) was categorised based on three proxy indicators: the type of living accommodation (rented or owned/mortgage), number of cars/vans/motorcycles in the household and the number of baths and/or showers and toilets in the home. Subjects were categorised as low level of material conditions if the subject was not homeowner and had at most one car, one bath shower and one toilet; high level of material conditions was considered if the subject had at least two cars and was either a homeowner or had two or more baths/showers or two or more toilets; all other people were classified in the intermediate level [29]. Education was assessed by the number of years in full-time education. Denture use (yes/no) was selected as the oral health–related covariate. APOE4 risk allele status was included as a genetic covariate, determined through genotyping to identify participants carrying at least one ε4 risk allele, given its well-established association with increased risk of cognitive decline and dementia. All covariate information used related to data collected at the 2001–2003 dental examination, which was considered the baseline for this study.

### 2.6 Statistical analysis

Comparisons of baseline characteristics were made between those who were cognitively normal at rescreening, those that developed MCI and those that developed dementia, using either analysis of variance (ANOVA) for continuous variables or a chi-square test for categorical variables. Continuous variables were summarised as mean (standard deviation) and categorical variables as n (%).

To facilitate statistical analysis and improve model stability in the multivariable analysis, a composite primary outcome of MCI/dementia was utilised. Multiple logistic regression analysis was conducted to determine odds ratios for the association between tooth loss (<20 teeth present) and MCI/dementia incidence. A series of pre-specified models was fitted to adjust for potential confounding variables. Model 1 adjusted for demographic and socioeconomic factors (age, baseline MMSE, education, and material conditions). Model 2 further included health-related variables (abdominal obesity, smoking, alcohol, diabetes, hypertension, and ACVD). Model 3, the fully adjusted model, added denture use and APOE4 allele status to account for oral health-related factors and genetic predisposition. In addition, a supplementary analysis examined the associations with MCI and dementia as separate outcomes (Supplementary information S1).

**Fig. 2:**
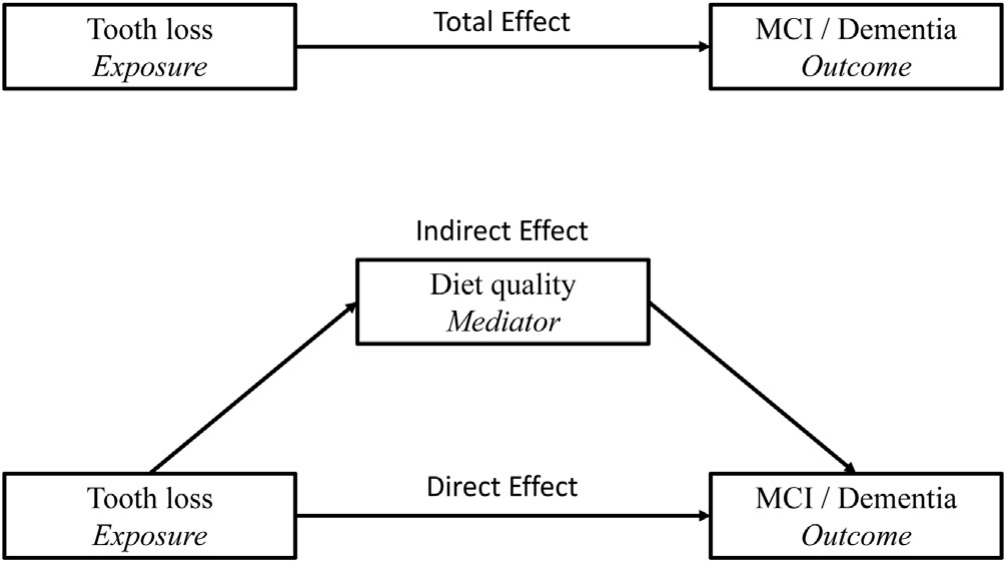
Depiction of mediation regression models. Models were adjusted for age, abdominal obesity, smoking, diabetes, hypertension, atherosclerotic cardiovascular disease, material conditions, education, denture use and APOE4 allele status.

A mediation analysis was conducted to understand the effect of diet diversity, using the Karlson-Holm-Breen (KHB) method [30]. Total, Direct, and Indirect Effects were calculated (Fig. 2), allowing calculation of the mediated proportion on the log-odds scale (proportion mediated = Indirect Effect/Total Effect). Models were adjusted for the same potential confounders as before (age, baseline MMSE, material conditions, education, abdominal obesity, smoking, alcohol, diabetes, hypertension, ACVD, denture use, and APOE4 allele status).

The level of statistical significance was set at *p* <  0.05. Analyses were performed using Stata release 17 (Stata Corp., College Station, TX).

## 3 Results

A total of 628 men who attended the baseline dental examination (2001–2003) with a cognitive impairment/baseline MMSE score ≥24/30, participated in the PRIME-COG rescreening (2016–2020) were included in the analysis (Fig. 1). The mean age of the men at baseline was 63.2 years (standard deviation [SD] 2.8), with a range of 58–71 years. The median follow-up time was 15.1 years (inter quartile range [IQR] 14.2–17.0). At cognitive rescreening, 485 men (77.2%) were assessed as cognitively normal, 112 men (17.8%) as having MCI, and 31 men (4.9%) as having dementia.

Baseline characteristics of the men by cognitive outcome are reported in Table 1. The mean number of teeth in men who were cognitively normal was 19.7 (SD 7.2), in those with MCI was 17.9 (SD 7.4), and in those with dementia was 16.5 (SD 8.1), *p* < 0.01. The percentage of men without a functional dentition (<20 teeth present) was 33.0% in the cognitively normal group, 48.2% in the MCI group and 64.5% in the dementia group, *p* < 0.001. There was an association across cognitive categories (cognitively normal, MCI, dementia) with smoking (*p* = 0.01): the proportion of never smokers decreased (46.8% vs. 30.4% vs. 29.0%), whilst the proportion of current smokers increased (13.0% vs 17.0% vs. 19.4%), respectively. Across cognitive outcomes there were also significant differences in proportions of men that initially had diabetes (*p* = 0.02) and those that hypertension (*p* < 0.01). Regarding socio-economic differences there were significant difference in proportions across categories of material conditions (*p* < 0.001), and across categories of years in education (*p* < 0.001). The mean Dietary Diversity Score was 6.3 (SD 1.0) in men who were cognitively normal, 5.8 (SD 2.0) in those with MCI, and 5.6 (SD 2.5) in those with dementia, *p* < 0.001.

**Table 1:** Characteristics of cohort studied by cognitive outcome (*n* = 628).

	Total cohort	Cognitively normal	MCI	Dementia	*p*
	*n* = 628 (100.0%)	*n* = 485 (77.2%)	*n* = 112 (17.8%)	*n* = 31 (4.9%)	
Number of teeth, mean (SD)	19.2 (7.3)	19.7 (7.2)	17.9 (7.4)	16.5 (8.1)	<0.01
Tooth loss (<20 teeth), *n* (%)	234 (37.3%)	160 (33.0%)	54 (48.2%)	20 (64.5%)	<0.001
Age, years, mean (SD)	63.2 (2.8)	63.1 (2.9)	63.6 (2.8)	63.8 (2.8)	0.18
Baseline MMSE, mean (SD)	28.5 (1.3)	28.6 (1.3)	28.2 (1.3)	27.9 (1.4)	<0.01
Waist circumference, cm, mean (SD)	94.4 (10.6)	94.0 (10.2)	95.0 (9.5)	98.5 (18.3)	0.06
Abdominal obesity, *n* (%)	112 (17.8%)	83 (17.1%)	20 (17.9%)	9 (29.0%)	0.24
Smoking, *n* (%)					
Never	270 (43.0%)	227 (46.8%)	34 (30.4%)	9 (29.0%)	0.01
Former	270 (43.0%)	195 (40.2%)	59 (52.7%)	16 (51.6%)	
Current	88 (14.0%)	63 (13.0%)	19 (17.0%)	6 (19.4%)	
Alcohol, *n* (%)	263 (42.0%)	201 (41.5%)	45 (40.2%)	17 (56.7%)	0.24
Diabetes, *n* (%)	37 (5.9%)	23 (4.7%)	13 (11.6%)	1 (3.2%)	0.02
Hypertension, *n* (%)	184 (29.3%)	127 (26.2%)	48 (42.9%)	9 (29.0%)	<0.01
ACVD, *n* (%)	55 (8.8%)	39 (8.0%)	12 (10.7%)	4 (12.9%)	0.47
Materials Conditions, *n* (%)					
Low	189 (30.1%)	131 (27.0%)	50 (44.6%)	8 (25.8%)	<0.001
Medium	162 (25.8%)	138 (28.5%)	20 (17.9%)	4 (12.9%)	
High	277 (44.1%)	216 (44.5%)	42 (37.5%)	19 (61.3%)	
Education, years, mean (SD)	11.9 (3.0)	12.3 (3.0)	10.5 (2.4)	11.3 (2.8)	<0.001
Education, categories, *n* (%)					
0–11 years	347 (55.3%)	246 (50.7%)	81 (72.3%)	20 (64.5%)	<0.001
12–14 years	166 (26.4%)	132 (27.2%)	26 (23.2%)	8 (25.8%)	
15–27 years	115 (18.3%)	107 (22.1%)	5 (4.5%)	3 (9.7%)	
Denture use, *n* (%)	290 (46.2%)	218 (44.9%)	54 (48.2%)	18 (58.1%)	0.33
APOE4 allele, *n* (%)	160 (25.5%)	110 (22.7%)	32 (28.6%)	18 (58.1%)	<0.001
Dietary Diversity Score, mean (SD)	6.2 (2.0)	6.3 (1.0)	5.8 (2.0)	5.6 (2.5)	<0.001

MCI: mild cognitive impairment; n: number; SD: standard deviation; MMSE: Mini-Mental State Examination; ACVD: atherosclerotic cardiovascular disease.

Multiple logistic regression analysis investigating independent variables and their association with the composite outcome of MCI/dementia is reported in Table 2. Men with tooth loss (<20 teeth present) had an OR = 2.18 (95% confidence interval [CI]: 1.49−3.18), *p* < 0.001 for an association with MCI/dementia incidence in the crude model. In the fully adjusted model (Model 3), this attenuated to an OR=2.06 (95% CI 1.20–3.55), *p* < 0.01. Confounding variables that were significantly associated with MCI/dementia incidence in the fully adjusted model also included baseline MMSE (*p* = 0.01), education (*p* < 0.001), hypertension (*p* < 0.01), and APOE4 allele (*p* < 0.01).

**Table 2:** Multiple logistic regression investigating independent variables and their association with MCI/dementia composite outcome (*n* = 628).

	Crude model	Model 1	Model 2	Model 3
	OR (95% CI)	OR (95% CI)	OR (95% CI)	OR (95% CI)
Tooth loss (ref. ≥ 20 teeth)	2.18 (1.49−3.18)	1.76 (1.18−2.62)	1.62 (1.07–2.45)	2.06 (1.20–3.55)
Age (per year increase)		1.05 (0.98–1.12)	1.04 (0.97–1.12)	1.05 (0.98–1.13)
Baseline MMSE score (per unit increase)		0.83 (0.72−0.96)	0.82 (0.70–0.95)	0.82 (0.71–0.96)
Education (ref. ≥ 15 years)				
12−14 years		3.38 (1.48–7.70)	3.45 (1.49–7.98)	3.42 (1.47–7.93)
0−11 years		4.39 (2.02–9.55)	4.31 (1.96–9.47)	4.37 (1.98–9.63)
Material conditions (ref. High)				
Medium		0.56 (0.33–0.96)	0.55 (0.31–0.95)	0.58 (0.33–1.01)
Low		1.17 (0.75–1.83)	1.10 (0.70–1.75)	1.22 (0.76–1.96)
Abdominal obesity (ref. Not obese)			0.93 (0.56–1.55)	0.94 (0.56–1.58)
Smoking (ref. Never smoker)				
Former			1.56 (0.99–2.46)	1.50 (0.95–2.38)
Current			1.81 (0.98–3.33)	1.73 (0.93–3.22)
Alcohol (ref. ≤14 units per week)			1.02 (0.68–1.53)	1.01 (0.66–1.52)
Diabetes (ref. No)			1.40 (0.66–2.99)	1.47 (0.68–3.16)
Hypertension (ref. No)			1.86 (1.22–2.85)	1.83 (1.19–2.81)
ACVD (ref. No)			0.93 (0.47–1.81)	1.02 (0.52–2.03)
Denture use (ref. No)				0.65 (0.38–1.11)
APOE4 allele (ref. No)				1.94 (1.24–3.03)

OR: odds ratio; CI: confidence interval; ref: reference category; ACVD: atherosclerotic cardiovascular disease.

Finally, to assess the extent to which diet diversity, measured using a DDS, mediated the relationship between tooth loss and the composite outcome of MCI/dementia, a mediation analysis utilising the KHB method was performed. This method decomposed the Total Effect of tooth loss on MCI/dementia incidence into Direct and Indirect Effects, thereby quantifying the contribution of dietary diversity to the observed association (Fig. 2). Among the 527 men with DDS data, there was a Total Effect of OR = 1.82 (95% CI 1.00–3.34) after adjusting for covariates (Table 3). Controlling for diet diversity, the direct effect of tooth loss was reduced to a non-significant OR = 1.58 (95% CI 0.86–2.93), leaving an indirect effect of OR = 1.15 (95% CI 1.01–1.31) through the mediating pathway. The proportion mediated equated to 23% in the fully adjusted model.

**Table 3:** Karlson–Holm–Breen (KHB) mediation analysis of the effect of tooth loss on mild cognitive impairment/dementia outcomes through diet diversity (*n* = 527*).

	Crude model^a^				Fully adjusted model^b^		
	β	OR (95% CI)	*p*		β	OR (95% CI)	*p*
Total Effect	0.78	2.19 (1.43–3.36)	<0.001		0.60	1.82 (1.00–3.34)	0.05
Direct Effect	0.60	1.83 (1.18–2.83)	<0.01		0.46	1.58 (0.86–2.93)	0.14
Indirect Effect	0.18	1.20 (1.06–1.35)	<0.01		0.14	1.15 (1.01–1.31)	0.03

Proportion of Total Effect mediated = 0.18/0.78 = 23% (crude model) and 0.14/0.60 = 23% (fully adjusted model).

* Dietary data were available for 527 of cohort; OR: odds ratio; CI: confidence interval.

a No confounder adjustment.

b Adjusted for age, baseline MMSE score, abdominal obesity, smoking, alcohol, diabetes, hypertension, atherosclerotic cardiovascular disease, material conditions, education, denture use, and APOE4 allele status.

## 4 Discussion

The main finding of this longitudinal cohort study was that tooth loss, (<20 teeth present), was significantly associated with subsequent MCI/dementia incidence in a group of men from Northern Ireland over a 15-year follow-up period. Compared with having a functional dentition (≥20 teeth), tooth loss was associated with approximately a doubling in the odds (OR = 2.06 (95% CI 1.20–3.55), *p* < 0.01) of MCI/dementia incidence in the fully adjusted model. Diet quality, measured using a dietary diversity score, was found to be a partial mediator of the association between tooth loss and MCI/dementia incidence, accounting for 23% of the Total Effect.

The results of this study corroborate previously published findings from longitudinal studies that have shown independent associations between tooth loss and MCI/dementia risk in general [8,10]. However, relatively few longitudinal studies have explored the potential mediating role of diet, and specifically diet quality, in this relationship. Kiuchi et al. conducted a six-year prospective longitudinal study involving 35,744 older Japanese adults to investigate how tooth loss might increase dementia risk, utilising the KHB mediation method to examine multiple potential mediators [31]. Tooth loss was self-reported and, as in the current study, the subjects were categorised into two groups: <20 teeth and ≥20 teeth. Although self-reported measures provide valuable insights, they are less precise than dental examinations, which offer a more accurate assessment of dental status. The study focused on weight loss and reduced fruit and vegetable intake as dietary mediators. Fruit and vegetable intake mediated 4.44% of the association in men and 8.45% in women. Although the magnitude of mediation was smaller than reported in the current study, the findings similarly emphasise the potential importance of diet quality, particularly fruit and vegetable intake, as a significant mediator in the tooth loss and cognition pathway. Mameno et al. conducted a nine-year longitudinal study of 293 Japanese older adults to examine the relationship between tooth loss, dietary intake, and cognitive function [32]. Dental status was assessed by registered dentists, and dietary intake was measured using a validated dietary history questionnaire. The study identified green and yellow vegetables, meat, and dairy products as key dietary components linking tooth loss to cognitive decline. Generalised estimating equations revealed that the intake of green and yellow vegetables and meat had a stronger influence on cognitive function than the number of teeth itself. While the focus on specific dietary components contrasts with the broader dietary diversity score (DDS) used in the current study, both highlight the potential role of diet quality in mediating the relationship between oral health and cognition.

A number of cross-sectional studies have also investigated the mediating role of diet; however, the cross-sectional design limits the ability fully to explore mediation pathways compared to longitudinal studies. Nonetheless, meaningful comparisons can still be drawn with the current study, particularly in highlighting key dietary factors linked to the relationship between tooth loss and cognitive function. Li et al. examined the relationship between tooth retention and cognitive function in a cross-sectional study of 6634 multi-ethnic older adults in Western China [33]. Nutritional status was assessed using the Mini Nutritional Assessment Short Form, which provides a broad evaluation of malnutrition risk and categorises participants as normal nutrition, at risk of malnutrition, or malnourished. Mediation analysis showed that nutritional status explained 40.7% of the association between tooth retention and improved cognitive outcomes. The smaller mediation proportion observed in the current study may reflect differences in sensitivity of the measurement tools, with the Mini Nutritional Assessment Short Form capturing a broader spectrum of malnutrition risk compared to the narrower focus on food variety provided by the DDS. Chen et al. (2024) investigated the role of pro-inflammatory dietary patterns in the relationship between tooth loss and cognitive function in a cross-sectional analysis of 1009 older U.S. adults from the National Health and Nutrition Examination Survey (NHANES) dataset [34]. The study utilised the Dietary Inflammatory Index (DII) and found that participants with fewer teeth consumed more inflammatory foods and scored lower on cognitive tests. Mediation analysis revealed that inflammatory dietary patterns accounted for 11% to 16% of the association between tooth loss and cognitive decline. While the DII focuses on the inflammatory properties of dietary intake, the DDS used in the current study assesses food variety, highlighting distinct but complementary aspects of dietary quality.

Dietary diversity has been widely recognised as a critical factor in mitigating cognitive decline, primarily due to the varied intake of essential nutrients it promotes. Studies indicate that poor dietary diversity is associated with an increased risk of cognitive impairment and decline, while higher dietary diversity scores are associated with better memory and slower cognitive decline, particularly in older populations [35]. While dietary diversity indices offer a practical measure of overall diet quality, their utility may be limited by the lack of specificity regarding the types and proportions of foods consumed. Nutrient-specific approaches targeting omega-3 fatty acids, vitamin B12, and antioxidants have shown promise, as these nutrients are directly linked to neuroprotective and anti-inflammatory pathways [36]. Biomarker-driven assessments, such as levels of carotenoids, tocopherols, and omega-3 indices, provide more precise insights into the bioavailability and efficacy of dietary components [37]. These biomarkers offer an advantage by capturing individual variations in metabolism and absorption, potentially providing a stronger link to cognitive outcomes. Emerging evidence from metabolomics studies has also identified dietary-derived metabolites associated with cognitive decline, highlighting the role of specific foods and metabolic pathways in cognitive aging [38]. Although dietary diversity remains a robust and holistic measure, integrating targeted dietary pattern approaches for brain health and biomarker assessments could enhance our understanding and optimisation of dietary strategies for preventing cognitive decline.

From an oral health intervention perspective, denture use has long been a standard approach for individuals with extensive tooth loss, aiming to restore oral function, improve dietary quality, and enhance overall well-being. In this study, including denture use as a confounding variable in the statistical model suggested some protective effect (OR = 0.65, 95% CI 0.38–1.11), although the finding did not reach statistical significance. The complexities of these issues are reflected in the literature, with some studies demonstrating improvements in dietary quality and nutrient intake attributable to dentures, while others report limited or no benefit, often pointing to poorly fitted dentures or inconsistent use [39,40]. Furthermore, emerging evidence indicates that denture use may confer broader benefits, including potential cognitive advantages, by facilitating adequate mastication and possibly enhancing cerebral blood flow [[41], [42], [43]]. Nevertheless, other studies report inconclusive results, highlighting the importance of comprehensive oral rehabilitation, including pre-prosthetic treatments, in achieving overall health gains [44]. Notably, such cognitive benefits appear contingent on denture functionality, with poorly fitted or unused dentures potentially exacerbating nutritional deficiencies and compounding cognitive decline [45]. Consequently, future research should prioritise evaluating denture functionality and related factors to elucidate the true impact of denture use on both diet and cognitive outcomes.

The strengths of this study include the homogeneity of the sample: White Western European men. Ethnicity has previously been shown to influence dementia risk [46], tooth loss [47], and diet diversity [48]. Therefore, restricting the study to a single ethnic group can reduce confounding arising from population-level differences in genetics, healthcare access, and cultural dietary habits. The limited age range of men at baseline (mean 63.2 [SD 2.8] years) also helps minimise confounding arising from wide variations in mid-to-late-life risk exposures that might otherwise obscure the associations under investigation. Due to the design of the PRIME study with its main aim to investigate risks factors for ACVD, we were also able to make use of a range of other data on potential confounding factors in the current study (such as smoking status, alcohol, anthropometric measurements, co-morbidity status, education, and socio-economic variables). The longitudinal design of the study, with a median 15-year follow-up, helps limit reverse causality by establishing a clearer temporal relationship between exposure and outcome. A further strength lies in the use of calibrated dental assessments, alongside the use of validated neurocognitive measures, ensuring greater accuracy in evaluating both oral health and cognitive function. To the authors’ knowledge, the present study is the first study investigating the mediating effect of diet diversity specifically on the relationship between tooth loss and cognitive decline.

There are several limitations to this study. Firstly, although the homogeneity of the sample (Western European men) minimised certain confounding factors, it also constrains the generalisability of the findings to other populations, including women and individuals from diverse ethnic backgrounds. Secondly, although the lengthy follow-up period enhanced the study’s ability to capture long-term cognitive outcomes, it introduced attrition bias as the number of participants decreased from 1558 men in 2001–2003 to 714 at the final follow-up, reducing to 628 once study exclusion criteria were applied. Nearly one third of the men died during the study observation period, and men who were too ill, resided in care facilities, or were withdrawn by their families—often due to cognitive problems—were also less likely to have participated in the cognitive follow up, potentially attenuating the observed associations. Thirdly, the reliance on a FFQ for the dietary assessment brings inherent limitations [49]. Although the FFQ captures broad dietary habits, it may not reflect participants’ full dietary repertoire, leading to possible under- or over-reporting, particularly among older adults prone to recall bias. In addition, its generic food categories do not clarify whether poor oral health specifically affects the consumption of certain fruits or vegetables, thus overlooking finer nuances of nutrient intake important for cognitive health. Furthermore, because the dietary assessment was undertaken alongside the cognitive rescreening, it did not account for dietary change that may have occurred during the 15-year interval since the oral assessments took place. Fourthly, measuring tooth loss only at baseline (2001–2003) may not fully capture the dynamic nature of dental changes over time, possibly underestimating the cumulative impact of tooth loss on diet quality and subsequent cognitive outcomes. Fifthly, we defined functional dentition as the presence of twenty or more teeth, consistent with previous research [50]. However, small numbers of participants with fewer teeth limited our ability to explore more granular tooth count categories. In addition, data on occlusal status, tooth position, and functional units were not collected. Finally, although we adjusted for key confounders such as age, education, material conditions, and genetic risk, the complex nature of both cognitive trajectories and oral health determinants raises the possibility of unmeasured confounding. Moreover, the discrepancy in regression coefficients between the unadjusted and adjusted models suggests potential interdependencies among variables, possibly reflecting additional confounding factors or underlying mediation pathways.

## 5 Conclusion

In conclusion, tooth loss was associated with increased MCI/dementia incidence over a 15-year observation period among older men from Northern Ireland. This relationship was partially mediated by dietary diversity, highlighting the important pathway linking oral health, diet quality, and cognitive outcomes. These findings also highlight the importance of preventive measures to maintain oral health and reduce tooth loss, which could potentially mitigate its downstream effects on diet quality and cognitive function. Future research should explore longitudinal changes in oral health and dietary habits, assess the functional quality of dentures and other forms of tooth replacement, and investigate specific nutrient intakes in relation to cognitive trajectories.

## Sources of funding

The PRIME study was supported by grants from the 10.13039/501100000274British Heart Foundation (PG/14/9/30632) and the HSC NI Public Health Agency (RRG/3282/05). The cognitive follow up of the cohort (PRIME-COG) was supported by a grant from the Dunhill Medical Trust (R514/1116).

## Data availability statement

The data supporting the findings of this study are available on request from the corresponding author. The data are not publicly available due to privacy or ethical restrictions.

## CRediT authorship contribution statement

**Lewis Winning:** Conceptualization, Formal analysis, Methodology, Writing - original draft. **Danielle Logan:** Methodology, Writing - review & editing. **Claire T. McEvoy:** Methodology, Writing - review & editing. **Dominic Farsi:** Methodology, Writing - review & editing. **Gareth J. McKay:** Methodology, Writing - review & editing. **Christopher C. Patterson:** Methodology, Writing - review & editing. **Peter Passmore:** Writing - review & editing, Funding acquisition. **Clive Holmes:** Writing - review & editing, Funding acquisition. **Gerard J. Linden:** Conceptualization, Investigation, Writing - review & editing, Funding acquisition. **Bernadette McGuinness:** Conceptualization, Project administration, Writing - review & editing, Funding acquisition.

## Declaration of competing interest

The authors declare that they have no known competing financial interests or personal relationships that could have appeared to influence the work reported in this paper.
