# Supplementary material for: Tooth loss, diet quality, and cognitive decline: A 15-year longitudinal study
Source: J Nutr Health Aging. 2025 Jun 27;29(9):100620. doi: 10.1016/j.jnha.2025.100620 (PMC12268072; doi:10.1016/j.jnha.2025.100620)
Supplement: Supplementary file 1 [file mmc1.docx]

# Supplementary information

To further investigate the associations between tooth loss and cognitive outcomes, a multinomial logistic regression analysis was conducted to estimate relative risk ratios (RRRs) for incident mild cognitive impairment (MCI) and dementia separately, using 'normal cognition' as the reference category (**Table S1**). This approach facilitates the identification of distinct associations between tooth loss and each cognitive outcome. Models were adjusted for the same co-variates used in the main analysis (age, baseline MMSE score, education, material conditions, abdominal obesity, smoking, alcohol, diabetes, hypertension, ACVD, denture use, and APOE4 allele status). It's important to note that the number of incident dementia cases (n=31) was relatively small. Including multiple covariates in the models with such a limited number of events may increase the risk of overfitting, potentially compromising the stability and generalisability of the estimates. Therefore, while the fully adjusted model in this supplemental analysis provides a comprehensive view, the results should be interpreted with caution, considering the potential limitations due to sample size.

## **Table S1.** Multinomial logistic regression investigating tooth loss’s association with cognitive outcomes (*n*=628)

| Cognitive outcome | Crude model  RRR (95% CI) | *p* | Fully adjusted model*  RRR (95% CI) | *p* |
| --- | --- | --- | --- | --- |
| Cognitively normal (n=485) | (base outcome) |  | (base outcome) |  |
| MCI (n=112) |  |  |  |  |
| Tooth loss (ref. ≥20 teeth) | 1.93 (1.07–3.49) | 0.03 | 1.91 (1.06–3.45) | 0.03 |
| Dementia (n=31) |  |  |  |  |
| Tooth loss (ref. ≥20 teeth) | 3.46 (1.19–10.03) | 0.02 | 3.05 (1.06–8.83) | 0.04 |
| *Notes.*  RRR: Relative Risk Ratio; CI: Confidence Interval; ref: reference category.  *Adjusted for: age, baseline MMSE score, education, material conditions, abdominal obesity, smoking, alcohol, diabetes, hypertension, and atherosclerotic cardiovascular disease, denture use, and APOE4 allele status. | | | | |
